# Postoperative spontaneous coronary artery dissection after major abdominal oncological surgery: a case report

**DOI:** 10.1093/jscr/rjag968

**Published:** 2026-11-04

**Authors:** Lamia S Alsubaie, Hesham Saida, Nayef Alzahrani

**Affiliations:** College of Medicine, King Saud University, Ash Shaikh Hasan Ibn Abdullah Al Ash Shaikh Street, King Saud University District, Riyadh 12372, Saudi Arabia; Department of Anesthesiology, King Abdulaziz Medical City, Ministry of National Guard Health Affairs, Prince Mutaib bin Abdullah bin Abdulaziz Road, Ar Rimayah District, Riyadh 11426 Saudi Arabia; Department of Surgery, King Abdulaziz Medical City, Ministry of National Guard Health Affairs, Mutaib bin Abdullah bin Abdulaziz Road, Ar Rimayah District, Riyadh 11426, Saudi Arabia

**Keywords:** spontaneous coronary artery dissection, ST-elevation myocardial infarction, postoperative complications, pulmonary embolism, sigmoid colon adenocarcinoma

## Abstract

Spontaneous coronary artery dissection (SCAD) is an uncommon non-atherosclerotic cause of acute coronary syndrome that can present as ST-elevation myocardial infarction (MI) and is particularly challenging because postoperative thrombosis and bleeding risks coexist. We report the case of a 44-year-old woman who underwent major abdominal oncologic surgery and developed a minor pulmonary embolism treated with anticoagulation. On postoperative day 4, she developed acute chest pain with ST-segment elevation. Emergency coronary angiography showed a long lesion involving the second diagonal branch, consistent with SCAD, with otherwise normal coronary arteries. She was managed conservatively. Her course was complicated by right JP drain-site bleeding, which required permanent heparin discontinuation and blood transfusion. This case illustrates SCAD as a cause of postoperative ST-elevation MI and the difficulty of balancing thrombosis and bleeding risk after major surgery.

## Introduction

Spontaneous coronary artery dissection (SCAD) is a rare cause of acute coronary syndrome, caused by separation of the coronary arterial wall leading to intramural hematoma formation or luminal compression. It occurs most often in young to middle-aged women [1, 2]. SCAD may present with unstable angina, non-ST elevation myocardial infarction (MI), ST-elevation MI, ventricular arrhythmia, or sudden cardiac death [1, 3]. It is particularly hard to recognize after surgery, since postoperative patients have several alternative explanations for these findings [1, 4]. Management is equally difficult: acute coronary syndrome pathways typically call for anticoagulant and antiplatelet therapy, but these can worsen bleeding risk soon after major surgery [3]. We report a case of SCAD presenting as ST-elevation MI after major abdominal oncologic surgery, complicated by pulmonary embolism (PE) and postoperative drain-site bleeding.

## Case report

A 44-year-old woman with hypertension, dyslipidemia, and gastroesophageal reflux disease presented with sigmoid colon adenocarcinoma and a suspicious liver lesion. Her case was discussed at a multidisciplinary tumor board meeting, and the consensus was to proceed with surgical resection. She underwent exploratory laparotomy with sigmoidectomy and primary anastomosis, liver segment 3/2 resection, para-aortic lymph node dissection, cholecystectomy, and right oophorectomy. Estimated blood loss was 700 ml, with no intraoperative complications. Final pathology showed pT4aN1a moderately differentiated sigmoid colon adenocarcinoma. The resected liver lesion, para-aortic lymph nodes, and right ovary were negative for malignancy. Perioperative analgesia consisted of intrathecal morphine (200 mcg) and fentanyl (15 mcg), followed by bilateral rectus sheath block using 15 ml of 0.25% bupivacaine on each side. Postoperative pain was well controlled.

Postoperatively, she was monitored in the intensive care unit (ICU), extubated successfully, remained clinically stable and was started on enoxaparin 40 mg daily for thromboprophylaxis. On postoperative day 2, the patient developed shivering, abdominal tenderness, and tachycardia. A contrast-enhanced computed tomography (CT) of the abdomen was performed, which showed expected postoperative changes; CT pulmonary angiography, obtained for the tachycardia, revealed segmental/subsegmental minor PE involving the upper lobes, and heparin infusion was started.

On postoperative day 4 she developed acute central chest pain radiating to the left shoulder, with nausea and diaphoresis. Electrocardiogram (ECG) showed normal sinus rhythm with ST-segment elevation in leads V1 to V3 (Fig. 1). Troponin-I increased from less than 10 ng/L preoperatively to 13764.3 ng/L. Emergency coronary angiography via right radial access showed a long lesion extending from the ostium to the distal segment of the second diagonal branch, consistent with SCAD, with no significant disease in the remaining coronary arteries (Fig. 2). No percutaneous coronary intervention was performed. Echocardiography showed preserved left ventricular systolic function and lower limb venous Doppler ultrasound was negative for deep venous thrombosis.

**Figure 1:**
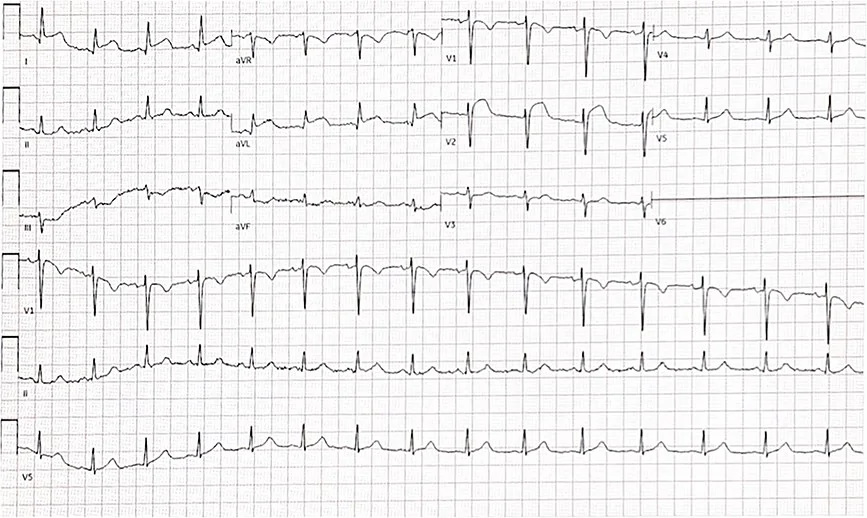
Twelve-lead ECG obtained during postoperative symptoms showing normal sinus rhythm with ST-segment elevation in the anterior leads.

**Figure 2:**
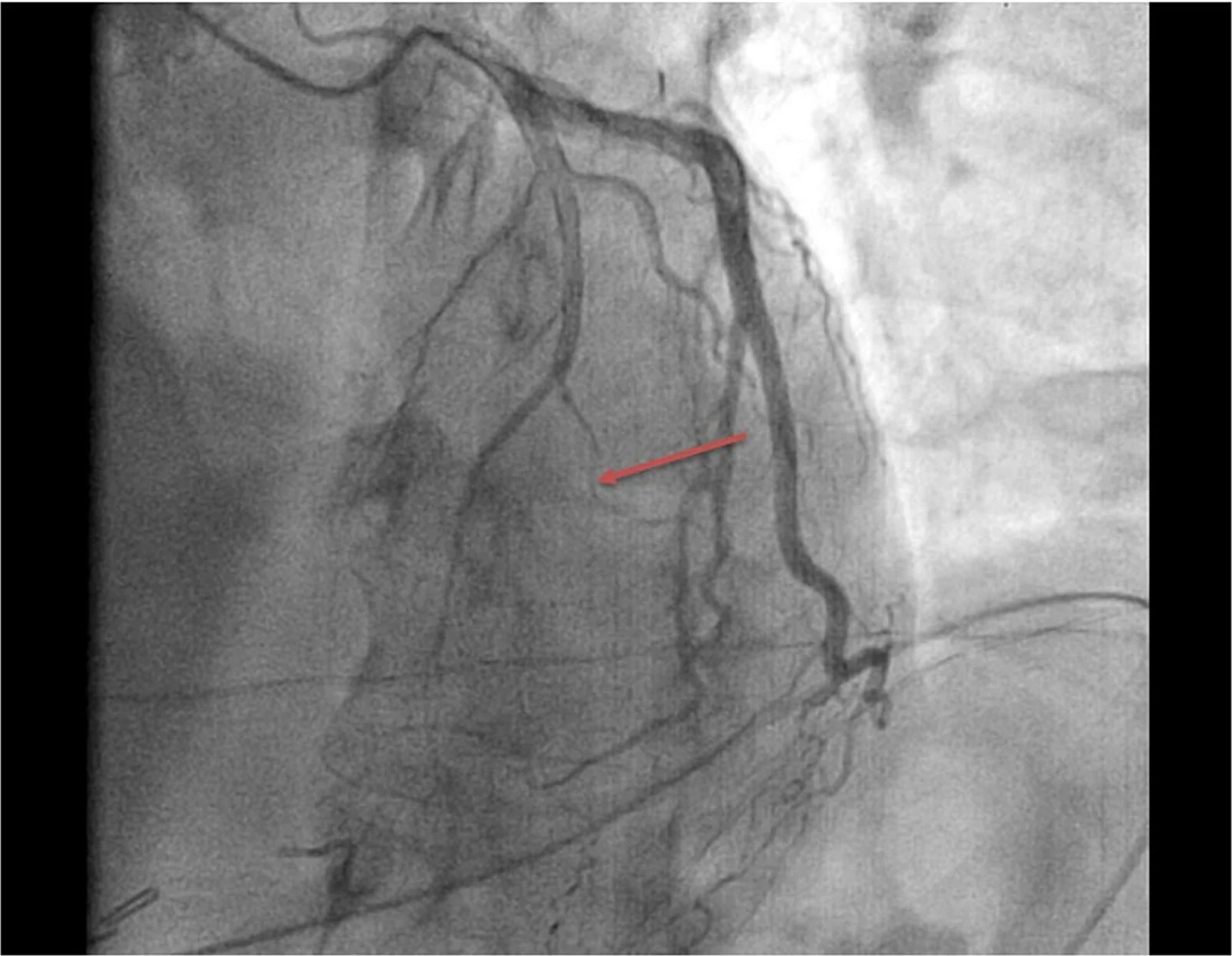
Coronary angiographic image of the left coronary circulation showing a long, narrowed lesion extending from the ostium to the distal segment of the second diagonal branch. The arrow indicates the affected vessel.

The initial cardiology plan included heparin infusion and dual antiplatelet therapy. However, this plan was revised after the patient developed bleeding from the right JP drain site with radiological evidence of active extravasation. The bleeding was managed conservatively; heparin was permanently discontinued, clopidogrel was stopped, and she received 3 units of packed red blood cells. Aspirin was continued once daily and beta-blocker therapy was optimized. Enoxaparin 40 mg twice daily was resumed for the PE once it was safe to do so. She had no recurrent chest pain and was discharged after a 17-day hospital stay on enoxaparin for 30 days. She remained symptom-free at follow-up.

## Discussion

Postoperative SCAD was diagnosed based on ischemic symptoms, ST-segment elevation, elevated troponin, and the angiographic finding.

Postoperative SCAD has been reported in a pregnancy-associated case following post-partum hemorrhage, in a case triggered during prone spinal instrumentation, and in a case following bilateral mastectomy for breast cancer [5–7]. This case differs from these reports in occurring after extensive abdominal oncologic surgery and in the antithrombotic dilemma it created: the patient needed anticoagulation for a newly diagnosed PE and SCAD but then developed drain-site bleeding requiring transfusion, so treatment of embolism had to be balanced against the risk of recurrent ischemia and further bleeding. To our knowledge, this is among the few reported cases of postoperative SCAD complicated simultaneously by acute PE requiring anticoagulation and clinically significant postoperative bleeding, highlighting the complexity of antithrombotic management in this setting.

Surgical stress itself has been reported as a trigger for SCAD across these settings [7]. A broader postoperative response is a more plausible trigger than uncontrolled pain.

Conservative management is generally preferred for stable SCAD without ongoing ischemia, left main involvement, or high-risk anatomy, which applied in this case, so no percutaneous intervention was performed. Antithrombotic therapy had to be individualized rather than following a fixed protocol: the initial acute coronary syndrome approach was revised once SCAD was identified and bleeding occurred, with heparin and clopidogrel stopped, beta-blockers along with aspirin continued, and enoxaparin used for the PE. This required coordination between cardiology, surgery, and interventional radiology. Clinicians should consider SCAD in postoperative patients who develop acute chest pain, ST-segment elevation, and troponin elevation. This case demonstrates the difficulty of managing postoperative SCAD in the presence of PE and active drain-site bleeding. Conservative management and individualized antithrombotic therapy led to a favorable short-term outcome.
